# Modulating Gradients in Regulatory Signals within Mesenchymal Stem Cell Seeded Hydrogels: A Novel Strategy to Engineer Zonal Articular Cartilage

**DOI:** 10.1371/journal.pone.0060764

**Published:** 2013-04-16

**Authors:** Stephen D. Thorpe, Thomas Nagel, Simon F. Carroll, Daniel J. Kelly

**Affiliations:** 1 Trinity Centre for Bioengineering, Trinity Biomedical Sciences Institute, Trinity College Dublin, Dublin, Ireland; 2 Department of Mechanical and Manufacturing Engineering, School of Engineering, Trinity College Dublin, Dublin, Ireland; University of Rochester, United States of America

## Abstract

Engineering organs and tissues with the spatial composition and organisation of their native equivalents remains a major challenge. One approach to engineer such spatial complexity is to recapitulate the gradients in regulatory signals that during development and maturation are believed to drive spatial changes in stem cell differentiation. Mesenchymal stem cell (MSC) differentiation is known to be influenced by both soluble factors and mechanical cues present in the local microenvironment. The objective of this study was to engineer a cartilaginous tissue with a native zonal composition by modulating both the oxygen tension and mechanical environment thorough the depth of MSC seeded hydrogels. To this end, constructs were radially confined to half their thickness and subjected to dynamic compression (DC). Confinement reduced oxygen levels in the bottom of the construct and with the application of DC, increased strains across the top of the construct. These spatial changes correlated with increased glycosaminoglycan accumulation in the bottom of constructs, increased collagen accumulation in the top of constructs, and a suppression of hypertrophy and calcification throughout the construct. Matrix accumulation increased for higher hydrogel cell seeding densities; with DC further enhancing both glycosaminoglycan accumulation and construct stiffness. The combination of spatial confinement and DC was also found to increase proteoglycan-4 (lubricin) deposition toward the top surface of these tissues. In conclusion, by modulating the environment through the depth of developing constructs, it is possible to suppress MSC endochondral progression and to engineer tissues with zonal gradients mimicking certain aspects of articular cartilage.

## Introduction

Adult articular cartilage consists of three separate structural zones; the superficial tangential, middle and deep zones. This depth dependent composition and organisation is fundamental to the normal physiological function of articular cartilage [1], [2]. Not only does cell morphology and arrangement change with depth, but each zone has distinct extra-cellular matrix (ECM) composition, architecture and mechanical properties. The dominant load carrying structural components of the ECM are collagen (∼75% tissue by dry weight) and proteoglycan (20%–30% tissue by dry weight), the concentrations of which vary with depth from the articular surface [1], [3], [4]. Collagen content is highest in the superficial zone, decreasing by ∼20% in the middle and deep zones [1], [3]. Proteoglycan content is lowest at the surface, increasing by as much as 50% into the middle and deep zones [3], [4]. The zonal composition and structural organisation of the ECM determine the biomechanical properties which also vary through the tissue depth; such that the compressive modulus increases from the superficial zone to the deep zone [2], [5], [6], while the tensile modulus decreases from the superficial surface to the deep zone [7].

An on-going challenge in the field of articular cartilage regeneration is the attainment of this stratified zonal structure. Classical tissue engineering approaches focus primarily on forming homogeneous tissues by embedding chondrocytes or stem cells in various scaffolds and do not attempt to mimic the organised zonal architecture of articular cartilage. One approach toward this aim is to utilise chondrocytes from specific zones of articular cartilage in the corresponding regions of an engineered construct [8]–[10]. It has been shown that chondrocytes from different zones demonstrate different biosynthetic activities [9], [10]. Layering such zonal chondrocytes in a photo-polymerising hydrogel has been shown to result in increased sulphated glycosaminoglycan (sGAG) accumulation in the bottom of the construct, although collagen content was also significantly higher in the bottom when compared to the top [10]. Another approach to engineering zonal cartilage is to vary biomaterial properties such as pore size [11], stiffness [12], [13] or composition [14], [15] through the depth of the scaffold or hydrogel. For example, combining layers of 2% and 3% agarose leads to zonal differences in the initial mechanical properties of the construct, however chondrocyte matrix elaboration in such bi-layered constructs was inferior to that in uniform 2% agarose [12]. Further improvements were observed with the application of dynamic compressive strain, or seeding zone specific chondrocytes into layered agarose hydrogels [13], [16]. While promising, there are potential limitations associated with such an approach, including the development of a distinct boundary between gel layers which could delineate as a result of shear stress [17]. Furthermore, isolation of chondrocytes from separate zones of articular cartilage can be difficult, particularly in damaged and diseased human tissue where the zonal differences are less distinct and potential biopsies are limited in size.

An alternative strategy to engineering zonal cartilage is to attempt to recapitulate aspects of the tissue micro-environment which may be responsible for the creation of depth-dependent properties in articular cartilage during development and maturation. In this respect, undifferentiated mesenchymal stem cells (MSCs) could prove to be a more appropriate cell source, as when provided with suitable stimuli they may differentiate into chondrocytes with specific zonal phenotypes. For example, low oxygen tension, a characteristic of avascular articular cartilage, has been shown to enhance chondrogenesis of mesenchymal stem cells (MSCs) [18]–[25]. In addition to oxygen, it has long been proposed that mechanical signals guide the differentiation of mesenchymal stem cells [26]–[30]. Hydrostatic pressure has been shown to promote a chondrogenic phenotype [31]–[34], enhancing collagen and sulphated glycosaminoglycan (sGAG) accumulation [35] and increasing the mechanical stiffness [36] of cartilaginous grafts engineered using MSCs encapsulated in agarose hydrogels. Dynamic compressive strain, another key component of the mechanical environment of articular cartilage, has also been shown to promote MSC chondrogenesis [30], [37]–[42]; positively modulating the functional development of cartilaginous constructs engineered using MSCs [43]. It has also been demonstrated that intermittent cyclic tensile strain applied to MSC seeded constructs increases collagen accumulation [44], [45].

The objective of this study was to engineer a cartilaginous construct with native-like zonal composition using MSCs by controlling both the oxygen tension and mechanical environment thorough the depth of the developing tissue. In an attempt to create a gradient in oxygen tension through the depth of the construct mimicking that in normal articular cartilage [46], the bottom halves of MSC seeded agarose constructs were radially confined; limiting oxygen transport into this region of the construct. Furthermore, by subjecting these radially confined constructs to dynamic compression it is possible to modulate the mechanical environment throughout the depth of the tissue, with higher levels of fluid pressure in the bottom of the construct and greater strains across the top of the construct. It is hypothesised that such a depth dependent microenvironment will lead to the development of zonal cartilage tissues with a composition mimicking that of normal articular cartilage.

## Materials and Methods

### Cell Isolation and Expansion

Porcine MSCs were isolated and maintained as previously described [42]. Animals were bred and raised for food and not research purposes and were not subject to any procedures prior to their sacrifice, hence no specific ethical approval was required for this study. Briefly, mononuclear cells were isolated from the femora of 4 month old pigs (∼50 kg) within 2 hours of sacrifice and plated at 10×10^6^ mononuclear cells per 75 cm^2^ culture flask (Nunclon; Nunc, VWR, Dublin, Ireland) allowing colony formation. MSCs were maintained in high-glucose Dulbecco’s modified eagle medium (4.5 mg/mL D-Glucose; hgDMEM) supplemented with 10% foetal bovine serum (FBS), penicillin (100 U/mL)-streptomycin (100 µg/mL) (all Gibco, Invitrogen, Dublin, Ireland) and amphotericin B (0.25 µg/mL; Sigma-Aldrich, Arklow, Ireland). Cultures were washed in Dulbecco’s phosphate buffered saline (PBS) after 72 hrs. When ∼75% confluent, MSCs were re-plated at 5×10^3^ cells/cm^2^ and expanded to passage two in a humidified atmosphere at 37°C and 5% CO_2_.

### Agarose Hydrogel Encapsulation and Construct Confinement

**Figure 1:**
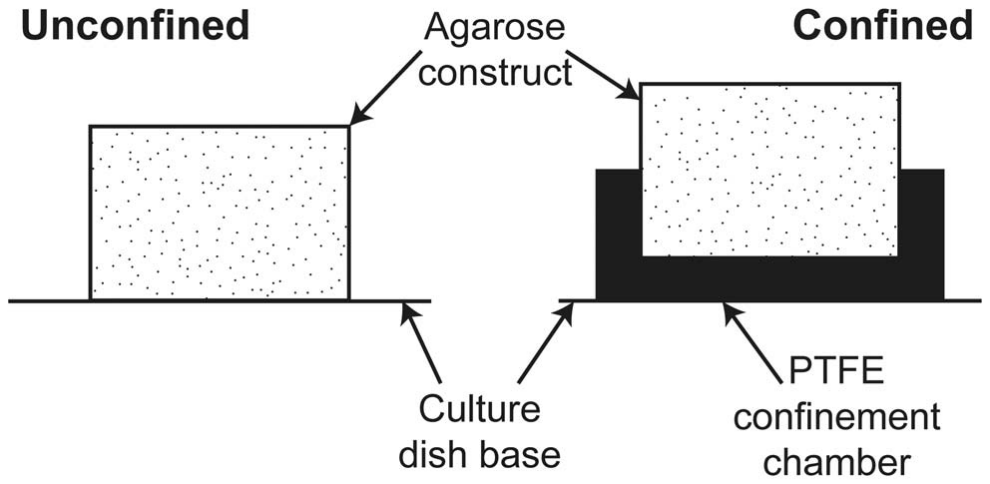
Experimental design. Constructs were press-fitted into custom made PTFE wells such that the bottom 2 mm of the construct thickness was confined.

MSCs were suspended in defined chondrogenic medium consisting of hgDMEM supplemented with penicillin (100 U/mL)-streptomycin (100 µg/mL) (both Gibco), 0.25 µg/mL amphotericin B, 100 µg/ml sodium pyruvate, 40 µg/mL L-proline, 1.5 mg/mL bovine serum albumin, 4.7 µg/mL linoleic acid, 1× insulin–transferrin–selenium, 50 µg/mL L-ascorbic acid-2-phosphate, 100 nM dexamethasone (all Sigma-Aldrich) and 10 ng/mL TGF-β3 (Pro Spec-Tany TechnoGene Ltd., Rehovot, Israel). This cell suspension was mixed with agarose (Type VII; Sigma-Aldrich) in PBS at a ratio of 1∶1 at approx. 40°C, to yield a final agarose concentration of 2% and a cell density of either 20×10^6^ cells/mL or 50×10^6^ cells/mL. The agarose-cell suspension was cast between stainless steel plates, one of which was overlaid with a patterned PDMS layer, allowed cool to 21°C for 30 min., and cored to produce cylindrical constructs (Ø 6 mm×4 mm thickness) which were patterned on one surface. Constructs remained patterned side up throughout culture and were maintained in ∼1 mL chondrogenic medium per 1×10^6^ cells/day with medium exchanged every 3 or 4 days and sampled for biochemical analysis. Either directly after fabrication or at day 21 of culture, constructs were press-fitted into custom made PTFE confinement chambers (Fig. 1) where they remained for the outstanding culture duration.

### Dynamic Compression Application

Dynamic compressive loading was applied as described previously [42] to constructs from day 21 to day 42 of culture. Unconfined intermittent dynamic compression (DC) was carried out in an incubator-housed, dynamic compression bioreactor and consisted of a sine wave of 10% strain amplitude superimposed upon a 1% pre-strain, with a 0.01 N per construct preload at a frequency of 1 Hz for 4 hours/day, 5 days/week.

### Mechanical Testing and Analysis of Physical Parameters

On removal from culture, construct diameter and wet weight (ww) were recorded. Constructs were mechanically tested in unconfined compression between impermeable platens using a standard materials testing machine (Bose Electroforce 3100; Bose Corporation, Gillingham, UK) as previously described [47]. A preload of 0.01 N was applied to ensure that the construct surface was in direct contact with the impermeable loading platens. Stress relaxation tests were performed consisting of a ramp displacement of 0.025%/s up to 10% strain, which was maintained until equilibrium was reached (∼30 minutes). This was followed by a dynamic test where cyclic strain amplitude of 1% (10%–11% total strain) was applied for 10 cycles at 1 Hz. Samples were subsequently sliced into top and bottom regions using a custom built rig and each region was tested simultaneously on separate, randomly assigned material testing machines (Zwick Roell Z005; Zwick Testing Machines Ltd., Herefordshire, UK) as above.

### Biochemical Constituents

After mechanical testing of top and bottom construct regions, the wet weight (ww) of each was recorded and the constructs frozen at −85°C for further analyses. The biochemical content of constructs was assessed as previously described [42]. Samples were digested with papain (125 µg/ml) in 0.1 M sodium acetate, 5 mM L-cysteine HCl, 0.05 M EDTA (all Sigma-Aldrich), pH 6.0 at 60°C under constant rotation for 18 hours. DNA content was quantified using the Hoechst Bisbenzimide 33258 dye assay (Sigma-Aldrich) as previously described [48]. The sulphated glycosaminoglycan (sGAG) content was quantified using the dimethylmethylene blue dye-binding assay (Blyscan; Biocolor Ltd., Carrickfergus, Northern Ireland). Total collagen content was determined by measuring orthohydroxyproline via the dimethylaminobenzaldehyde and chloramine T assay [49]. A hydroxyproline-to-collagen ratio of 1∶7.69 was used [50]. Cell culture media was analysed for sGAG and collagen secreted.

### Histology and Immunohistochemistry

Constructs were fixed in 4% paraformaldehyde, paraffin embedded and sectioned at 5 µm to produce a cross section perpendicular to the disc face. Sections were stained for sGAG with 1% alcian blue 8 GX in 0.1 M HCl, for collagen with picro-sirius red and for calcific deposition with 1% alizarin red (all Sigma-Aldrich). Immunohistochemistry was performed as described previously [42]. Sections were enzymatically treated with chondroitinase ABC (Sigma-Aldrich) in a humidified environment at 37°C. Slides were blocked with goat serum (Sigma-Aldrich) and sections were incubated for 1 hour with a primary antibody diluted in blocking buffer specific to either collagen type I (1∶400, 1 mg/mL), collagen type II (1∶100; 1 mg/mL), collagen type X (1∶200; 1.4 mg/mL) (all Abcam, Cambridge, UK) or proteoglycan-4 (PRG4; 1∶200; 1 mg/mL) (Sigma-Aldrich). After washing in PBS, sections were incubated for 1 hour in the secondary antibody, anti-mouse IgG biotin antibody produced in goat (1∶200; 2 mg/mL; Sigma-Aldrich). Colour was developed using the Vectastain ABC kit followed by exposure to peroxidase DAB substrate kit (both Vector Laboratories, Peterborough, UK). Immunofluorescent staining was employed for collagen type X, which involved permeabilisation of the cell membrane with 0.1% Triton-X100. In place of colour development, sections were incubated with ExtrAvidin-FITC (1∶100; Sigma-Aldrich) for 1 hour, washed several times in PBS, nuclei counterstained with DAPI (1∶500; 1 mg/mL; VWR), and sections mounted using Vectashield (Vector Laboratories). Sections were imaged with an Olympus IX51 inverted fluorescent microscope fitted with an Olympus DP70 camera. Sections of porcine cartilage, ligament and/or growth plate were included as controls.

### Theoretical Prediction of Mechanical Environment within Agarose Constructs

To estimate the effect of semi-confinement on the mechanical environment within the construct, pore pressure and maximum principal strain were predicted for day 0 constructs. The loading protocol of the bioreactor was simulated with material properties derived from a sample specific fit to constructs mechanically tested at day 0. Cell-seeded constructs were modelled as fluid saturated porous media using a finite strain formulation applied previously to agarose in compression [51]. Surfaces in contact with media were modelled as free draining, while fluid flow was prohibited across surfaces in contact with the loading platens or the confinement well. Nonlinear permeability was modelled following Gu *et al.* [52]. Briefly, the solid matrix was modelled as nonlinearly viscoelastic based on a multiplicative decomposition of the deformation gradient into elastic and viscous parts *F = F_e_F_v_*. An evolution equation was defined for the viscous right Cauchy-Green tensor *C_v_*:
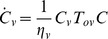
where *η_v_* is the viscosity function, *T_ov_* is the viscoelastic overstress and *C* is the right Cauchy-Green tensor [51], [53]. While the equilibrium free Helmholtz energy potentials where based on a Neo-Hookean formulation



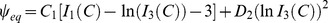
the viscoelastic overstresses where derived from the exponential potential



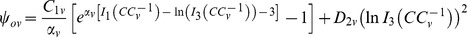



The equilibrium properties *C_1_* and *D_2_* were determined analytically from the relaxed part of the ramp and hold test. *D_2v_* was set to *C_1v_D_2_/C_1_*. The remaining parameters in the viscoelastic potential and the viscosity were fit to unconfined ramp and hold force relaxation curves using a differential evolution algorithm developed by Storn and Price [54]. For further details see Görke *et al.* [51].

### Measurement and Prediction of Oxygen Concentration within Agarose Constructs

Local oxygen concentration was assessed using implantable fibre optic oxygen micro-sensors (Microx TX; PreSens – Precision Sensing GmbH, Regensburg, Germany). Media was added to day 5 constructs seeded with 20×10^6^ cells/mL in confined and unconfined configurations so that the surface of the media was 1 mm above the top surface of the construct. The sensor tip was positioned at the media surface above the centre of the construct. Constructs were allowed equilibrate for ≥24 hours in an incubator at 18.5% oxygen prior to oxygen measurement. A linear actuator (NA0830; Zaber Technologies Inc., Vancouver, Canada) was used to control the movement of the sensor tip which was moved vertically downward along the construct axis at 1.8 µm/s to within ∼1 mm of the construct base; to avoid collision of the probe with the culture dish or confinement chamber. Oxygen concentration was sampled every 5 s.

**Figure 2:**
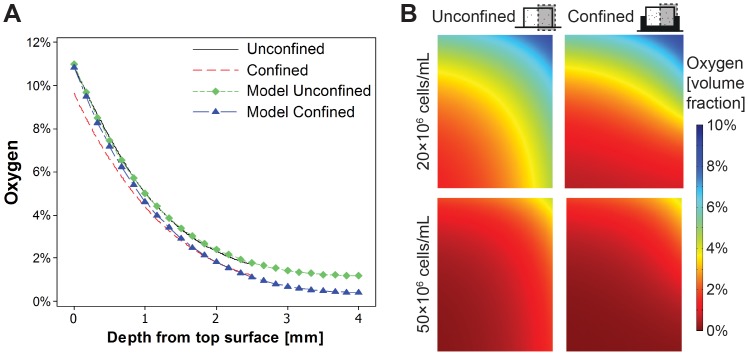
Oxygen tension is modulated by the cell seeding density and radial confinement. MSCs encapsulated in agarose at 20×10^6^ cells/mL were cultured for 4 days, at which point constructs were confined and allowed to equilibrate for 24 hours. (A) Oxygen concentration through the media and along the construct axis was measured for unconfined and confined constructs with representative samples of each presented (Exp). Model predictions were fit to experimental data obtained from the surface of the culture media through the depth of the construct. For clarity, only the fit through the construct is shown (Model). (B) Predicted gradients in oxygen volume fraction for unconfined and confined constructs with seeding densities of 20×10^6^ and 50×10^6^ cells/mL. Oxygen volume fraction corresponds to molar fraction (×10 pmol/mm^3^).

Oxygen concentration in the constructs was modelled using a diffusion-reaction type equation. The reaction term followed Michaelis-Menten kinetics:
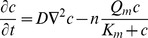
Here, *c* is the oxygen concentration, *D* the diffusion coefficient, *n* the cell density, *Q_m_* the maximum consumption rate and *K_m_* the concentration at half the maximum consumption rate. The diffusion coefficient in 2% agarose was determined using the Mackie and Meares relation



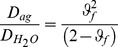
so that *D_ag_* = 2.77×10^−3^ mm^2^/s and *φ_f_* is the fluid phase volume fraction [55]. The cellular consumption value was set to *Q_m = _*13.5×10^−18^ mol/cell/s as determined by the model fit to the experimental data for unconfined constructs with 20×10^6^ cells/mL (Fig. 2A). This value was similar to previously reported values for MSC oxygen consumption while undergoing chondrogenic differentiation [56]. The Michaelis-Menten constant, *K_m_* was set to 5×10^−5^ µmol/mm^3^. The sensitivity of the simulation outcome to this value is very low. The construct was modelled as axisymmetric. Oxygen diffusion through the culture media was accounted for by setting *D_media_* = 3.0×10^−3^ mm^2^/s. The oxygen concentration at the media surface was prescribed as 185 µM, while at surfaces in contact with the bottom of the well, the confining chamber and the symmetry axis the flux was set to zero. The simulations were performed for cell concentrations of *n* = 20×10^6^ cells/mL and *n* = 50×10^6^ cells/mL.

### Statistical Analysis

Presented are results from one of two replicate studies with unique donors where *n* refers to the number of constructs analysed for each assay within a given replicate (*n* numbers provided in figure legends). Statistics were performed using MINITAB 15.1 software package (Minitab Ltd., Coventry, UK). Where necessary, a Box-Cox transformation was used to normalise data sets. Construct groups were analysed for significant differences using a general linear model for analysis of variance with factors of group, confinement, dynamic compression, construct region and interactions between these factors examined. Tukey’s test for multiple comparisons was used to compare conditions. Significance was accepted at a level of *p*≤0.05. Numerical and graphical results are presented as mean ± standard error. Statistical results displayed in figures are from the post hoc tests and represent differences between specific treatment groups. *p* values in the text may refer to either main effects or post hoc tests.

## Results

### Radial Confinement Spatially Alters the Pore Pressure and Tensile Strain within Agarose Hydrogels during Dynamic Compression

**Figure 3:**
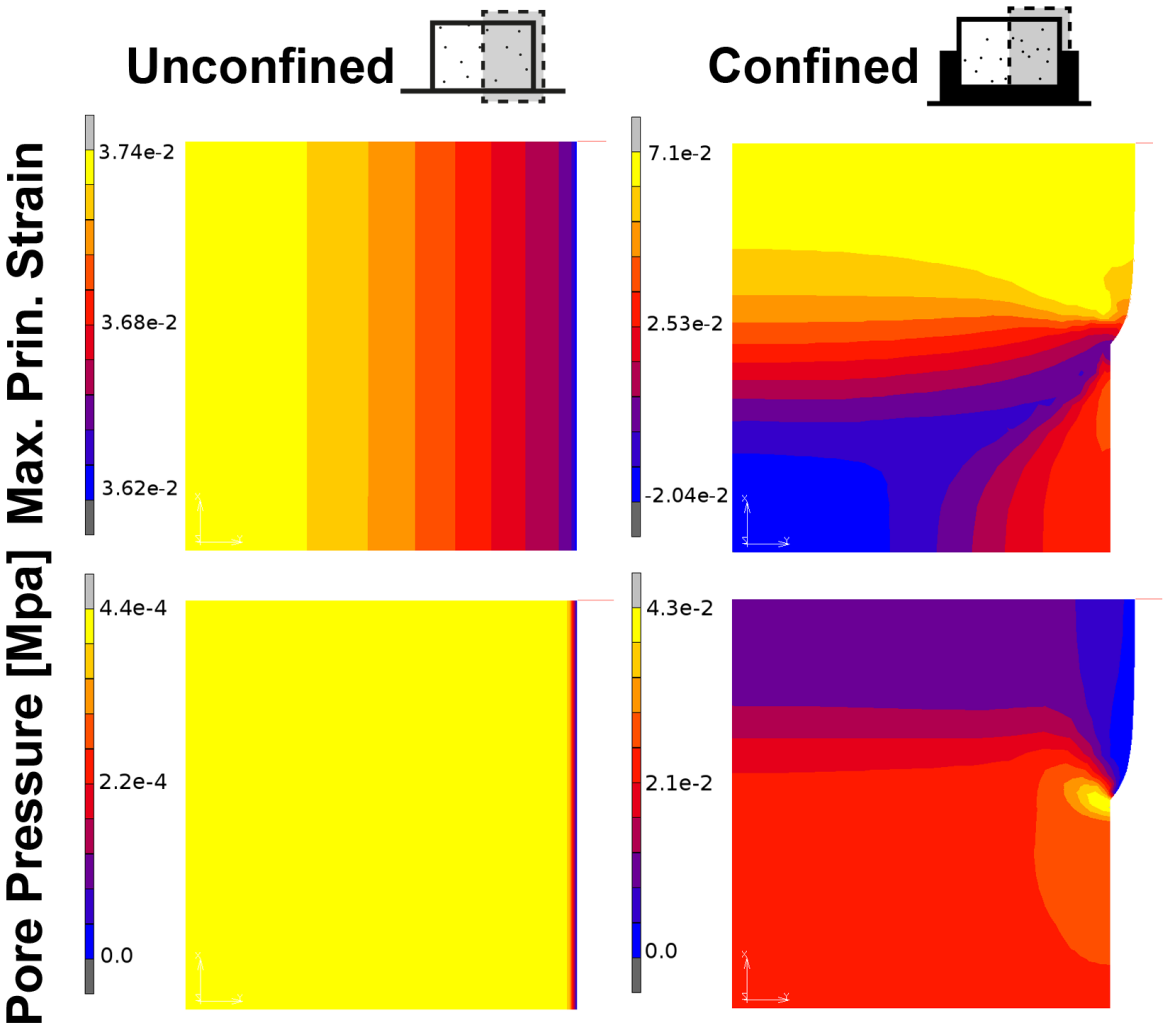
Partial radial confinement alters pore pressure and strain distribution under dynamic compression. Theoretical predictions of the maximum principle strain and pore pressure [MPa] for both unconfined and confined configurations during steady state dynamic compression at day 0.

The mechanical environment within an agarose hydrogel during dynamic compression was predicted for both unconfined and confined configurations (Fig. 1). A relatively homogenous strain environment is predicted within the unconfined construct while the confined constructs experience higher tensile strains in the top of the construct with predominantly compressive strains of lower magnitude present in the bottom (Fig. 3). The model predicts pore pressures in the bottom of the confined constructs which are about an order of magnitude greater than that in the unconfined configuration where a relatively homogenous pressure environment exists.

### Oxygen Tension within Engineered Tissues is Modulated by the Cell Seeding Density and Radial Confinement

The oxygen concentration along the construct axis was measured as a function of depth for constructs seeded at 20×10^6^ cells/mL (Fig. 2A). Due to cellular consumption, an oxygen gradient develops over time. For unconfined constructs sitting on the base of a dish (i.e. the unconfined free swelling constructs in this study), the oxygen concentration decreases towards the bottom centre; from 10.82% at the top surface to 1.70% at a depth of 2.5 mm from this top surface. Oxygen concentration at a depth of 2.5 mm from the top of the construct was lower in confined constructs when compared to unconfined controls; 1.20% vs. 1.70% respectively. This data was fit to a computational model to predict spatial gradients in oxygen concentration throughout constructs seeded with both 20×10^6^ cells/mL and 50×10^6^ cells/mL (Fig. 2B). Increasing the cell density to 50×10^6^ cells/mL accentuated these gradients in oxygen tension. Confinement led to further reductions in oxygen concentration and enlargement of the low oxygen region across the bottom of the construct; culminating in the development of a low oxygen region (with a minimum predicted value of 0.39%) at 20×10^6^ cells/mL and an anoxic region (approaching 0%) predicted at the 50×10^6^ cells/mL seeding density.

### Radial Confinement Enhances sGAG Accumulation in the Bottom of Engineered Cartilaginous Constructs

**Figure 4:**
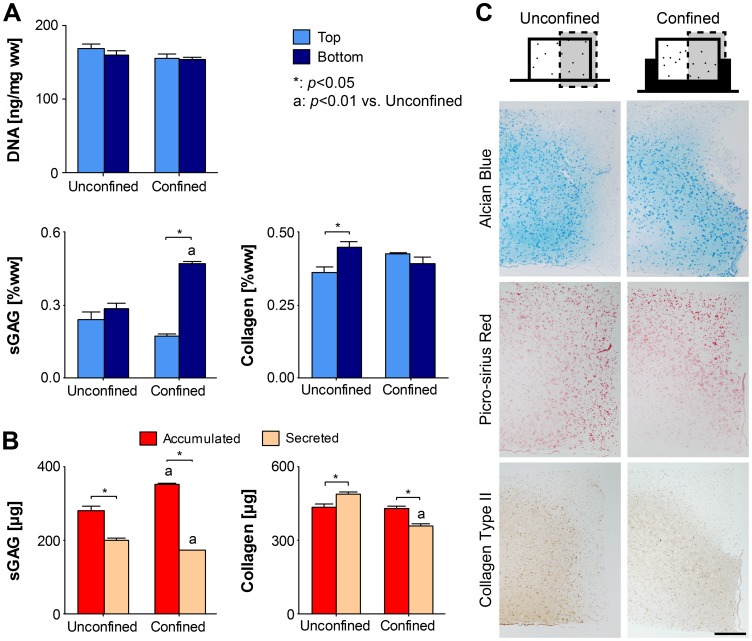
Radial confinement enhances sGAG accumulation in the bottom of engineered cartilaginous constructs. MSCs encapsulated in agarose at 20×10^6^ cells/mL were cultured for 21 days in unconfined or confined conditions. (A) The top and bottom regions of unconfined and confined free-swelling constructs were analysed for DNA, sGAG and collagen contents. (*n* = 4) (B) Total sGAG and collagen accumulated in the constructs (sum of top and bottom) and secreted to the media. Media data is presented as µg accumulated and secreted (*n* = 4) (C) Unconfined and confined constructs were stained with alcian blue for sulphated mucins, picro-sirius red for collagen and immunohistochemically for collagen type II. Representative full-depth half construct sections are shown as indicated. (*n* = 2) Scale bar 500 µm.

MSCs were encapsulated in agarose at 20×10^6^ cells/mL, confined, and cultured in free-swelling (unloaded) conditions to day 21 at which point the biochemical content was independently assessed within the top and bottom regions of the construct (Fig. 4A). No differences in DNA content were observed between the top and bottom of either confined or unconfined constructs. For unconfined constructs, sGAG content at day 21 did not change significantly with construct depth. However confinement led to an increase in sGAG in the construct bottom when compared to unconfined controls (0.470±0.009%ww vs. 0.283±0.024%ww; *p* = 0.0002); resulting in depth-dependent sGAG accumulation by day 21 (*p*<0.00005). When the media was analysed, it was revealed that confinement served to decrease sGAG secretion to the media (*p* = 0.004; Fig. 4B); in spite of this, total sGAG produced (accumulated plus secreted to media) was still greatest in confined constructs (527.95±4.536 µg vs. 485.348±13.595 µg; *p* = 0.0154). Collagen accumulation was also depth dependent in unconfined constructs at day 21 with greater collagen accumulation in the bottom (*p* = 0.0247; Fig. 4A). Confinement acted to nullify this non-cartilaginous zonal variation in collagen concentration, with no difference between the two regions. Analysis of the media revealed that while confinement did not affect collagen accumulated, it did reduce collagen secreted to the media, such that more collagen was synthesised in unconfined constructs (929.45±15.426 µg vs. 753.852±12.597 µg; *p*<0.0001; Fig. 4B).

Histological staining confirmed that spatial variations exist in the distribution of sGAG and collagen within constructs (Fig. 4C). In confined constructs, more intense alcian blue and collagen type II staining is evident towards the edge in the bottom (confined) region of the construct when compared to unconfined controls.

### Radial Confinement Coupled with Dynamic Compression Enhances Collagen Accumulation in the Top of the Construct

MSCs were encapsulated in agarose at 20×10^6^ cells/mL and cultured in unconfined free-swelling (unloaded) conditions for 21 days, at which point constructs were confined and dynamic compression applied from day 21 to 42. This delayed application of dynamic compression was motivated by our previous finding that dynamic compression application from day 0 inhibited chondrogenesis of MSCs [30], [42], [57], [58].

**Figure 5:**
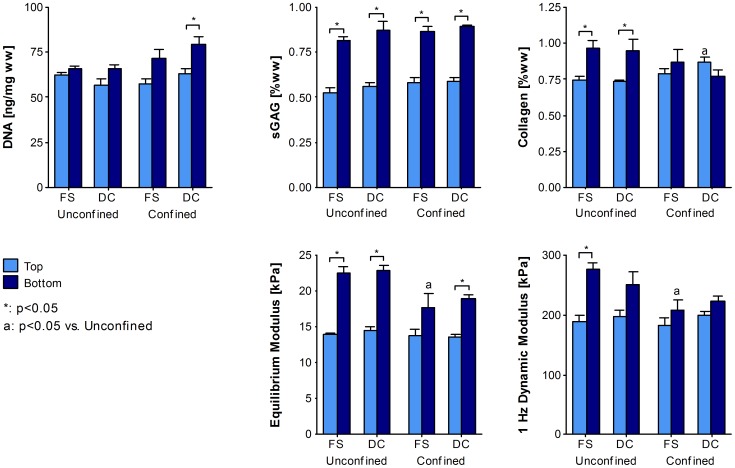
Radial confinement coupled with dynamic compression enhances collagen accumulation in the top of the construct. Agarose constructs containing MSCs at 20×10^6^ cells/mL were confined from day 21 to day 42 of culture while 10% dynamic compression was applied. The top and bottom regions of constructs were analysed for DNA, sGAG and collagen contents. Top and bottom regions of constructs were also mechanically tested for both the equilibrium modulus and the dynamic modulus at 1 Hz. FS: free-swelling; DC: dynamic compression. (*n* = 4).

While there was no difference in sGAG content between top and bottom construct regions for unconfined constructs at day 21, ensuring constructs remained the same way up over 42 days of culture did eventually lead to greater sGAG accumulation in the construct bottom when compared to the top (*p*<0.0001; Fig. 5). Confinement from day 21 did not further enhance sGAG accumulation in either region. When normalised to DNA content, only unconfined constructs exhibited a significant difference in sGAG/DNA with depth (Fig. S1). Neither dynamic compression nor confinement had a significant effect on total sGAG accumulation, although compression did increase sGAG secretion to the media (*p* = 0.0344; Fig. S1). By day 42 unconfined constructs also exhibited zonal variation in collagen content with greater accumulation in the bottom of the construct (*p* = 0.0016; Fig. 5). However, construct confinement again acted to reduce this zonal variation such that there was no longer a significant difference between top and bottom. Moreover, when confinement was combined with dynamic compression, this zonal gradient was reversed such that collagen content in the top of confined compressed constructs was greater than that in unconfined controls (0.868±0.033 vs. 0.736±0.011; *p* = 0.0252; Fig. 5). When normalised to DNA, collagen synthesis in the top of confined constructs was significantly higher than that in the bottom (*p* = 0.0382; Fig. S1). This change in relative collagen accumulation with depth was due both to an increase in collagen accumulation in the top of the construct, and a decrease in collagen accumulation in the bottom. Approximately half the total collagen produced was secreted to the media, with neither confinement nor dynamic compression having any significant effect (Fig. S1).

**Figure 6:**
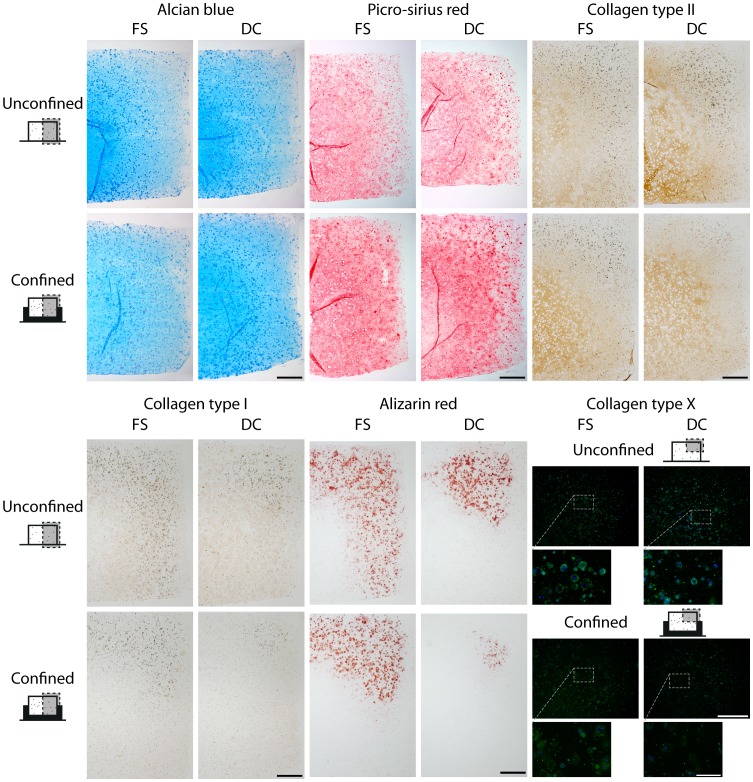
Radial confinement coupled with dynamic compression suppresses endochondral progression. Agarose constructs containing MSCs at 20×10^6^ cells/mL were confined from day 21 to day 42 of culture while 10% dynamic compression was applied. Constructs at day 42 were stained with alcian blue for sulphated mucins, picro-sirius red for total collagen, alizarin red for calcific deposition and immunohistochemically for collagen type I, collagen type II and collagen type X. Representative full-depth half construct sections are shown for all but collagen type X where a representative quarter section is shown in immunofluorescence. Collagen type I and collagen type II staining is indicated in brown, with calcific deposits evident in black. FS: free-swelling; DC: dynamic compression. (*n* = 2) Scale bar 500 µm. Inset scale bar 100 µm.

Confinement acted to increase the intensity of collagen staining in the top half of the construct when compared to unconfined controls; particularly in constructs subjected to dynamic compression (Fig. 6). Similar staining patterns were evident for collagen type II (Fig. 6). The combination of dynamic compression and confinement also led to a reduction in staining for collagen type I (Fig. 6). Dynamic compression reduced calcific deposition in unconfined constructs, as evident by reduced alizarin red staining (Fig. 6). A further reduction in alizarin red staining was observed when constructs were both confined and subjected to dynamic compression. Although collagen type X immunofluorescence was present in confined constructs, the combination of confinement and dynamic compression also led to a reduction in staining intensity (Fig. 6).

Though the equilibrium and dynamic moduli increased with time for all conditions (*p*<0.001), bulk construct mechanical properties were unaffected by confinement or dynamic compression at day 42 (data not shown). In agreement with sGAG zonal variation, the equilibrium modulus was greater in the bottom of constructs than the top (*p*<0.0001; Fig. 5). This region-specific increase in equilibrium modulus was greater in unconfined controls (*p* = 0.0008; Fig. 5).

### MSC Response to Extrinsic Signals is Dependent on the Cell Seeding Density

While cartilaginous constructs with a varying zonal composition can be engineered by controlling the environment through the depth of the developing construct, absolute levels of matrix accumulation were lower than native values. In an attempt to address this issue, MSCs were encapsulated in agarose at a higher seeding density of 50×10^6^ cells/mL and cultured in unconfined free-swelling (unloaded) conditions for 21 days, at which point constructs were confined and dynamic compression applied from day 21 to 42.

**Figure 7:**
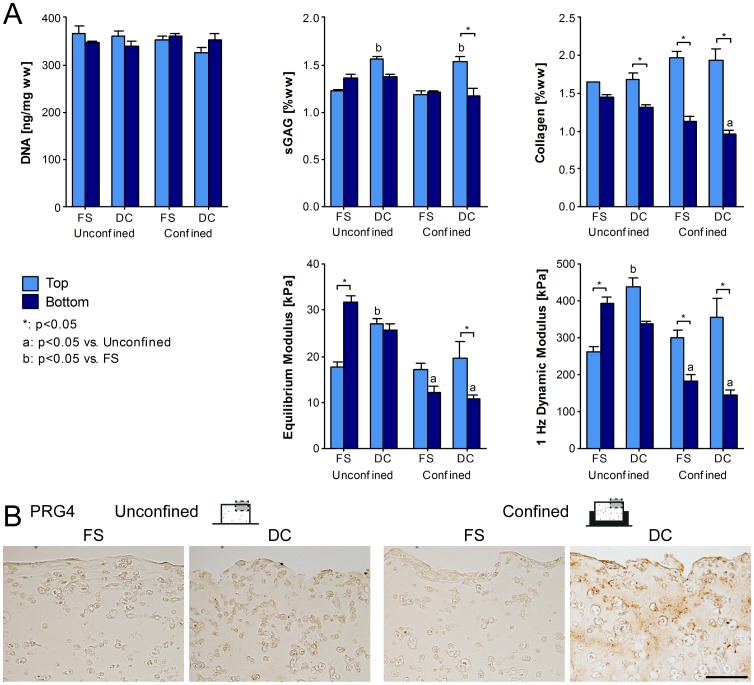
MSC response to extrinsic signals is dependent on the cell seeding density. Agarose constructs containing MSCs at 50×10^6^ cells/mL were confined from day 21 to day 42 of culture while 10% dynamic compression was applied. (A) The top and bottom regions of constructs were analysed for DNA, sGAG and collagen contents. Top and bottom regions of constructs were also mechanically tested for both the equilibrium modulus and the dynamic modulus at 1 Hz. (*n* = 4) (B) Constructs at day 42 were stained immunohistochemically for proteoglycan-4 (PRG4). FS: free-swelling; DC: dynamic compression. (*n* = 2) Scale bar 100 µm.

Dynamic compression increased total sGAG content for both confined and unconfined constructs compared to free swelling controls at day 42 (*p* = 0.0005; Fig. 7A). However this increase in sGAG occurred in the top of the construct (*p* = 0.0001); such that confinement combined with dynamic compression led to greater sGAG accumulation in the construct top compared to the bottom (1.538±0.040%ww vs. 1.170±0.092%ww; *p* = 0.0013; Fig. 7A). On analysis of culture media, confinement was seen to inhibit total sGAG produced (*p* = 0.0094; Fig. S2). Notably more sGAG was secreted to the media than retained within the construct (*p* = 0.0020). Confinement acted to increase collagen accumulation by day 42 in the construct top when compared with unconfined controls irrespective of the application of dynamic compression (*p* = 0.0137; Fig. 7). However, confinement led to an overall decrease in collagen content in the construct bottom in comparison to unconfined controls (*p* = 0.0026; Fig. 7, Fig. S2). On inclusion of collagen secreted to the media, it was evident that confinement at 50×10^6^ cells/mL also inhibited total collagen production (*p* = 0.0407; Fig. S2).

Although proteoglycan-4 (PRG4) staining was weak in constructs seeded with 20×10^6^ cells/mL, at 50×10^6^ cells/mL the combination of confinement and dynamic compression was observed to increase PRG4 staining at the top surface of constructs (Fig. 7B). Collagen staining decreased toward the core region of all constructs and was more heterogeneous than alcian blue staining (Fig. S3).

Dynamic compression acted to enhance both the equilibrium and dynamic modulus for the whole construct (*p*<0.05; data not shown). Regionally, this increase was only evident in the construct top, which was stiffer than the corresponding region in FS controls (*p*<0.05; Fig. 7). Confinement had a negative effect on whole construct stiffness (*p*<0.05); attributed to a decrease in both equilibrium and dynamic moduli in the bottom of confined constructs corresponding to lower matrix accumulation in this region (*p*<0.01; Fig. 7).

## Discussion

Significant developments have been made regarding the use of MSCs for functional cartilage tissue engineering [59]–[61]. However, attempts to engineer grafts with a zonal composition and organisation mimicking normal articular cartilage have been limited. Creating such zonal variations in tissue composition within engineered grafts may be critical to regenerating hyaline cartilage with a normal Benninghoff architecture [62]. Here we show that the depth dependent properties of cartilaginous grafts engineered using MSCs can be modulated through spatial alteration of the oxygen tension and the mechanical environment within the developing construct. MSCs were encapsulated in agarose hydrogel and confined up to half their thickness. This reduced oxygen levels in the bottom of the construct and when combined with dynamic compression, increased tensile strains across the top of the construct. These spatial changes in the local environment correlated with increased sGAG accumulation in the bottom of constructs, increased collagen accumulation in the top of the construct, and a near complete suppression of MSC hypertrophy and tissue calcification throughout the depth of the engineered tissue. Consequently, a tissue with depth dependent variation in sGAG, collagen and mechanical properties similar, but not identical to native articular cartilage was established. In an effort to increase extracellular matrix (ECM) accumulation and mechanical properties, constructs were seeded at a higher cell density (50×10^6^ cells/mL). This failed to instigate the creation of a native-like zonal composition in biochemical constituents and mechanical properties, demonstrating that the spatial environment within constructs seeded at very high densities was not conducive to engineering zonal constructs with cartilage-like properties.

Implantable fibre-optic oxygen micro-sensors and computational modelling were used to assess the spatial alterations in oxygen tension and the mechanical environment induced by confinement and dynamic compression respectively. Due to the uncertainty associated with material parameter identification for constructs later in culture where elaborated extracellular matrix may alter nutrient transfer and inhomogeneously alter construct mechanics, the simulations were performed for the initial cell seeded agarose constructs only. Despite this limitation, the measurement of oxygen availability and the finite element simulations provided insight into the spatial gradients in the mechanical environment and the oxygen tension throughout the construct and its dependence on cell density.

Initially, the effect of confinement was examined over 21 days. Confinement enhanced sGAG accumulation in the bottom (confined) region of the construct. One explanation for this was that confinement was simply acting to reduce secretion of ECM components into the media. While less sGAG was secreted from confined constructs, the total produced remained significantly higher with confinement (Fig. 4B), supporting the hypothesis that the low oxygen microenvironment predicted within the bottom of confined constructs may be enhancing chondrogenesis. Regions of faint alcian blue and collagen type II staining (Fig. 4C) in both confined and unconfined constructs also appeared to correlate with predicted regions of higher oxygen availability (Fig. 2B). While confinement lowers the availability of oxygen in the bottom of confined constructs, it could also act to reduce the availability of other regulatory factors such as TGF-β3 or glucose. However, in contrast to the abundance of data available in the literature supporting the argument that lower oxygen levels enhance chondrogenesis of MSCs [18]–[25], to the author’s knowledge, there is no data available in the literature demonstrating that lower levels of other factors such as TGF-β3 or glucose will enhance cartilage matrix specific ECM synthesis in bone marrow derived MSCs. While we believe the lower levels of oxygen within the bottom of confined constructs are acting to enhance chondrogenesis in this region of the construct, we cannot rule out the possibility that gradients in other factors may also be playing a role.

In contrast to the findings over the first three weeks of culture, confinement from day 21 to day 42 did not lead to enhanced sGAG accumulation. In fact, sGAG accumulation was concentrated in the bottom of both confined and unconfined constructs at day 42. Given that care was taken to prevent constructs from flipping over during culture (thereby ensuring that the top surface of the construct remained face upwards for the culture duration), a low oxygen region will naturally develop in the bottom of the construct due to cellular consumption (Fig. 2), which may explain why sGAG accumulation in the long term is higher in the bottom of both confined and unconfined constructs. It should be noted that the oxygen levels within the engineered tissue will also theoretically depend on the depth of media above the construct, and this should be considered when designing culture strategies to engineer zonal tissues.

When confinement was combined with dynamic compression, collagen accumulation increased in the top of the construct resulting in a depth-dependent zonal variation in both the biochemical composition and compressive properties of the engineered tissue. The increased levels of collagen synthesis in the top of mechanically stimulated confined constructs may be due to the higher levels of strain within this region of the engineered tissue (Fig. 3). Intermittent cyclic tension has specifically been shown to increase collagen content within MSC seeded constructs [44], [45]. In the present study, tensile strains across the top of dynamically compressed constructs were predicted to approximately double due to radial confinement (Fig. 3); perhaps implicating this stimulus in promoting the higher levels of collagen accumulation in this region of the construct. Interestingly, the combination of confinement and dynamic compression also acted to inhibit hypertrophy (as evident by reduced type X collagen staining) and calcification (as evident by reduced alizarin red staining) of the engineered tissue. We have previously demonstrated that a low oxygen tension supresses the endochondral phenotype of chondrogenically primed MSCs [25], and here we provide further support for the role of dynamic compression in ensuring the development of a stable chondrogenic phenotype [30], [63], suggesting that gradients in oxygen levels and mechanical cues may together regulate hypertrophy in developing articular cartilage. Although fluid pressures in the bottom of confined constructs were increased due to dynamic compression, this did not lead to a corresponding increase in sGAG accumulation in this region of the construct at either cell seeding density. This may be due to the fact that the pressure generated remains over an order of magnitude lower than that reported to elicit increases in chondrogenic gene expression for MSCs [31], [32], [35].

The effect of dynamic compression was also dependent on the cell seeding density. At a seeding density of 50×10^6^ cells/mL dynamic compression acted to enhance sGAG accumulation and the bulk construct mechanical properties in both unconfined and confined conditions. Long-term dynamic compression of MSC seeded agarose applied after 21 days of unloaded pre-culture has previously been shown to augment sGAG accumulation [42] and enhance construct stiffness [43]. This finding that MSC response to dynamic compression is dependent on cell seeding density has also recently been reported [63]. At the higher seeding density of 50×10^6^ cells/mL, it is possible that greater cell-cell contact and signalling may enhance MSC response to dynamic compression. It is also possible that a threshold level of ECM accumulation is required for dynamic compression to stimulate additional synthesis, as increasing the cell density was seen to increase total matrix accumulation. Démarteau *et al.* demonstrated that chondrocyte response to dynamic compression was positively correlated to the level of ECM accumulation [64]. Additionally, dynamic compression may facilitate enhanced transport of nutrients and other regulatory factors within the construct [65]–[67]. Increasing cell density acts to increase the severity of nutrient gradients due to cellular consumption in the construct periphery, which may be somewhat overcome through the application of dynamic compression.

At 50×10^6^ cells/mL, the combination of confinement and loading also led to an increase in PRG4 staining across the top of the engineered tissue, which may be due to the high levels of deformation present in this region of the construct. This protein is known to play an important role in joint lubrication, and its expression has previously been shown to increase with mechanical stimulation [68], [69].

While a construct with a zonal variation in mechanical properties and biochemical composition mimicking certain aspects of normal articular cartilage was achieved with a seeding density of 20×10^6^ cells/mL, this was not the case at the higher seeding density of 50×10^6^ cells/mL. Increasing the cell seeding density presumably increased consumption of oxygen and other solutes within the construct, resulting in a more acute decrease in nutrient availability away from the periphery. Confinement of constructs added to the severity of this gradient, such that an anoxic region was predicted in the bottom of confined constructs at the 50×10^6^ cells/mL seeding density (Fig. 2B); lower than that predicted within the deep zone of native articular cartilage [46]. It is expected that gradients similar to that predicted for oxygen will develop for other solutes such as ascorbate and glucose and that one, or a combination of these may compromise cell metabolism culminating in the reduced ECM production observed at 50×10^6^ cells/mL. While matrix accumulation was significantly reduced in regions of nutrient limitation, DNA content did not follow the same trend, with no differences between construct regions or conditions (Fig. 7A), suggesting that cells were viable but in a quiescent state. When cultured at low oxygen, MSCs have demonstrated a robust glycolytic potential [56], [70] which may explain the survival of these cells in this potentially anoxic environment. It has recently been shown that optimal nutrient supply is crucial to the production of functional cartilage matrix using MSCs [71]. Vascularised cartilage canals are present in developing cartilage [72], [73] and may provide a route for oxygen and nutrient supply. Mimicking such nutrient paths in engineered constructs [74], [75] may provide a means to overcome transport limitations in such tissues.

Recently, through incorporation of specific natural and synthetic components into polyethylene glycol hydrogels, it has been demonstrated that it is possible to direct MSC differentiation into zone-specific phenotypes [15]; and through layering of these zone-specific hydrogels, engineer a depth-dependent tissue [76]. This approach was successful in the induction of cartilage-like zonal variations in collagen type II, type X, and sGAG production. The strategy adopted in this study, namely to modulate gradients in biomechanical and biochemical signals within the developing tissue, also has the potential to induce cartilage-like zonal variations in ECM content in one cohesive construct. Furthermore, this system may better recapitulate the spatial patterns of regulatory cues determining articular cartilage organisation during postnatal development, and may prime the construct for *in vivo* implantation as it will be subject to similar environmental cues within a load bearing defect. By coupling this zonal approach with strategies that attempt to spatially regulate endochondral ossification within MSC seeded hydrogels [77], it may be possible to also engineer functional osteochondral grafts.

### Conclusion

Engineering cartilaginous grafts with structural composition and organisation is crucial to the long term repair of cartilage lesions. By controlling the oxygen tension and mechanical environment through the depth of the developing tissue, MSC differentiation was modulated such that a construct with depth-dependent sGAG and collagen content somewhat akin to that of articular cartilage was engineered. This paper represents a novel approach toward engineering an organ or tissue with zonal variations in biochemical composition and mechanical properties. While there are still challenges to be overcome in order to engineer a native-like zonal articular cartilage tissue, and whether such bioreactor systems are ultimately used to engineer cartilaginous grafts for the clinic remains an open question [78], the results of this study help us to elucidate how environmental factors regulate MSC differentiation and in this case, the development and organisation of articular cartilage; knowledge that will be central to developing new therapies for damaged and diseased joints.

## Supporting Information

Figure S1 **Radial confinement coupled with dynamic compression enhances collagen accumulation in the top of the construct.** Agarose constructs containing MSCs at 20×10^6^ cells/mL were confined from day 21 to day 42 of culture while 10% dynamic compressive strain was applied. The top and bottom regions of constructs were analysed for sGAG and collagen contents which were normalised to DNA content. sGAG and collagen accumulated within the construct and secreted to culture media was also measured. FS: free-swelling; DC: dynamic compression. Dynamic compression as a main effect led to enhanced sGAG secretion to the media; *p* = 0.0344. *n* = 4. *: *p*<0.05; **a**: p<0.05 vs. Unconfined.
(TIF)

Figure S2 **MSC response to extrinsic signals is dependent on the cell seeding density.** Agarose constructs containing MSCs at 50×10^6^ cells/mL were confined from day 21 to day 42 of culture while 10% dynamic compressive strain was applied. The top and bottom regions of constructs were analysed for sGAG and collagen contents which were normalised to DNA content. sGAG and collagen accumulated within the construct and secreted to culture media was measured. FS: free-swelling; DC: dynamic compression. *n* = 4. *: *p*<0.01; **a**: *p*<0.05 vs. Unconfined; **b**: *p*<0.05 vs. FS.
(TIF)

Figure S3 **MSC response to extrinsic signals is dependent on the cell seeding density.** Constructs at day 42 were stained with alcian blue for sulphated mucins, picro-sirius red for total collagen, and immunohistochemically for collagen type I and type II. Representative full-depth half construct sections are shown. FS: free-swelling; DC: dynamic compression. (*n* = 2) Scale bar 500 µm.
(TIF)
