# Supplementary figures and images for: Modulating Gradients in Regulatory Signals within Mesenchymal Stem Cell Seeded Hydrogels: A Novel Strategy to Engineer Zonal Articular Cartilage
Source: PLoS One. 2013 Apr 16;8(4):e60764. doi: 10.1371/journal.pone.0060764 (PMC3628868; doi:10.1371/journal.pone.0060764)

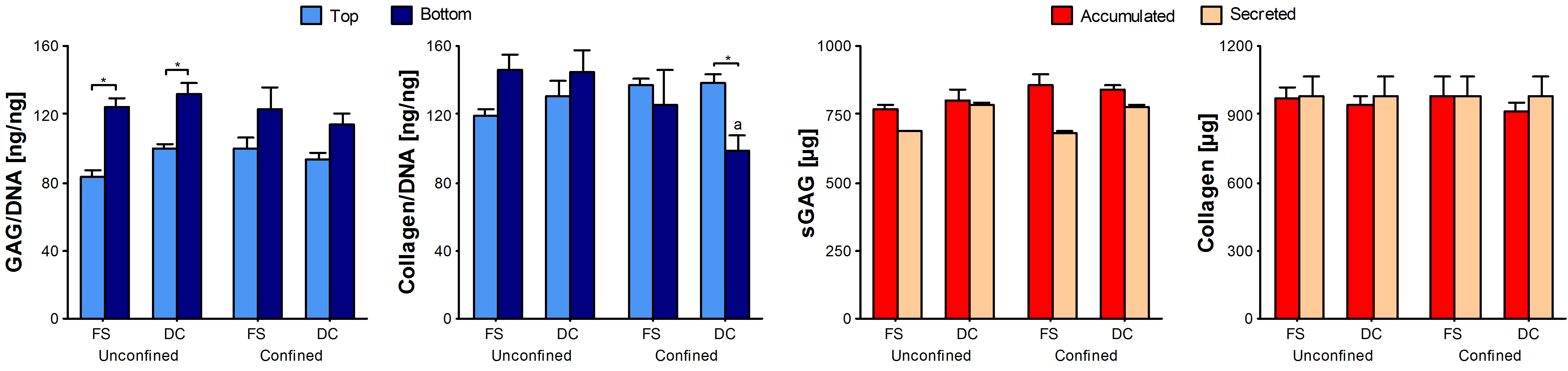

Supplement: Figure S1 — Radial confinement coupled with dynamic compression enhances collagen accumulation in the top of the construct. Agarose constructs containing MSCs at 20×106 cells/mL were confined from day 21 to day 42 of culture while 10% dynamic compressive strain was applied. The top and bottom regions of constructs were analysed for sGAG and collagen contents which were normalised to DNA content. sGAG and collagen accumulated within the construct and secreted to culture media was also measured. FS: free-swelling; DC: dynamic compression. Dynamic compression as a main effect led to enhanced sGAG secretion to the media; p = 0.0344. n = 4. *: p<0.05; a: p<0.05 vs. Unconfined. (TIF) [file pone.0060764.s001.tif]

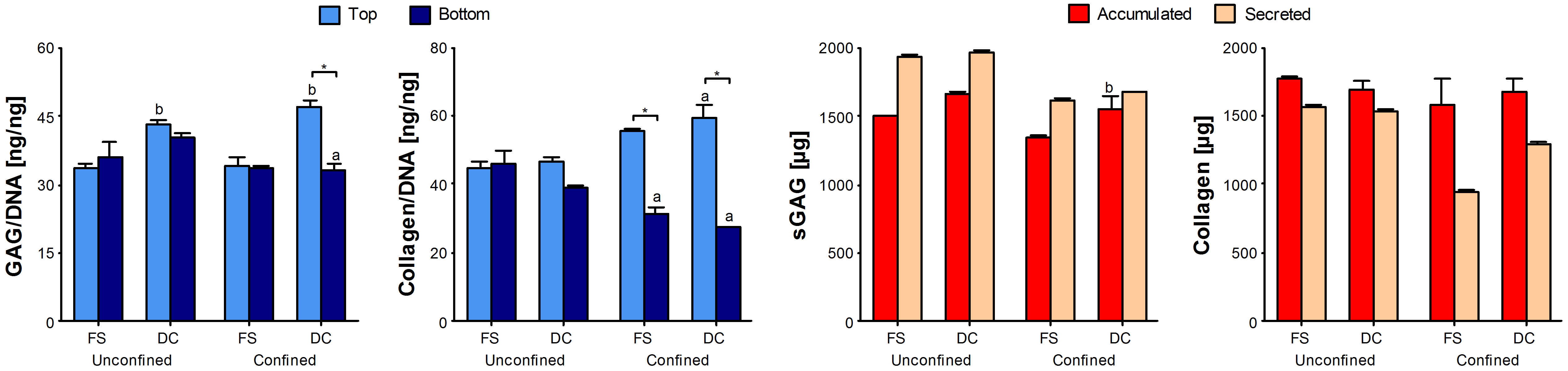

Supplement: Figure S2 — MSC response to extrinsic signals is dependent on the cell seeding density. Agarose constructs containing MSCs at 50×106 cells/mL were confined from day 21 to day 42 of culture while 10% dynamic compressive strain was applied. The top and bottom regions of constructs were analysed for sGAG and collagen contents which were normalised to DNA content. sGAG and collagen accumulated within the construct and secreted to culture media was measured. FS: free-swelling; DC: dynamic compression. n = 4. *: p<0.01; a: p<0.05 vs. Unconfined; b: p<0.05 vs. FS. (TIF) [file pone.0060764.s002.tif]

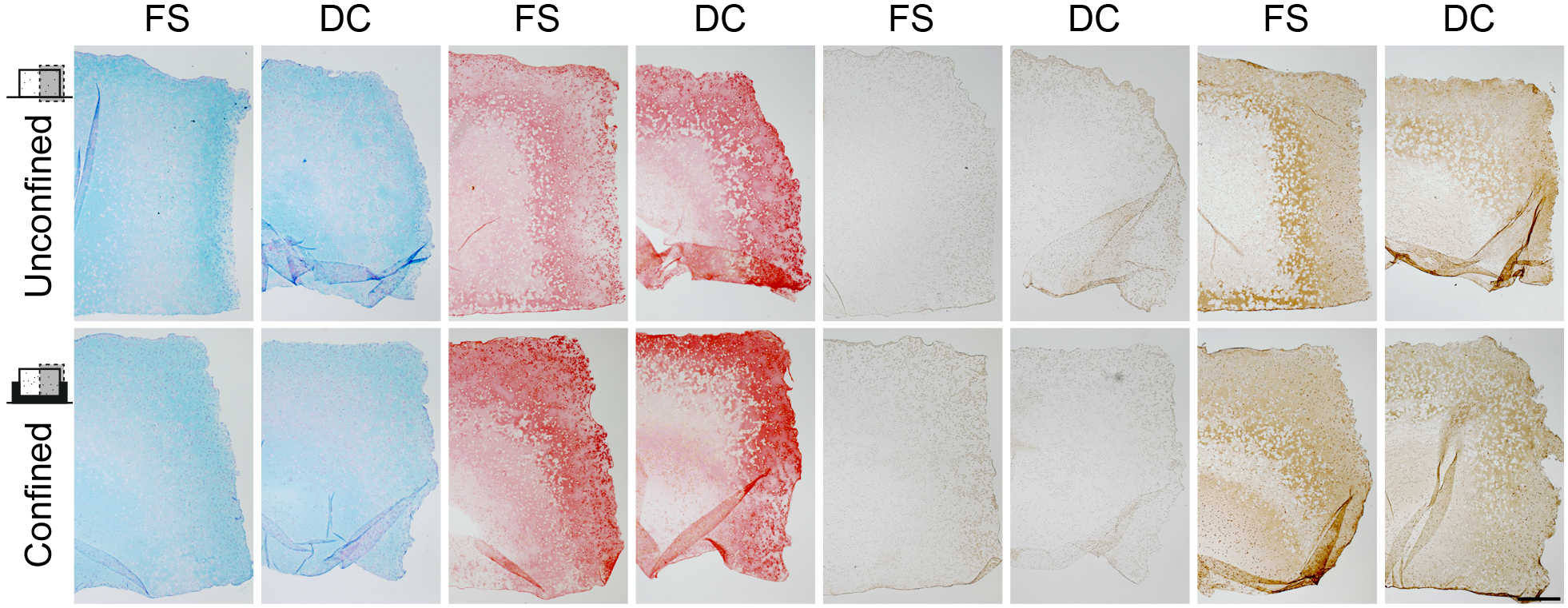

Supplement: Figure S3 — MSC response to extrinsic signals is dependent on the cell seeding density. Constructs at day 42 were stained with alcian blue for sulphated mucins, picro-sirius red for total collagen, and immunohistochemically for collagen type I and type II. Representative full-depth half construct sections are shown. FS: free-swelling; DC: dynamic compression. (n = 2) Scale bar 500 µm. (TIF) [file pone.0060764.s003.tif]
